# Innovating Social-Emotional Learning to Enhance Positive Engagement of Youth With Social Media: A Comprehensive Review of Why and How

**DOI:** 10.7759/cureus.70130

**Published:** 2024-09-24

**Authors:** Sajita Setia, Michelle Tichy, Frank Gilbert

**Affiliations:** 1 Executive Office, Transform Medical Communications Limited, Auckland, NZL; 2 Executive Office, Transforming Life LLC, Wilmington, USA; 3 Department of Psychology/Educational Psychology, Alfred University, New York, USA; 4 Department of Psychology, Norfolk State University, Norfolk, USA

**Keywords:** adolescents, educational psychology, mental health, positive psychology, public health, social media, youth

## Abstract

The rising mental health burden among youth, exacerbated by excessive social media use and other risks associated with digital engagement, necessitates urgent and innovative preventive solutions. The existing research supports the notion that smartphones and social media significantly impact youth mental health, advocating for age restrictions to mitigate worsening psychological distress in the young.This comprehensive narrative literature review synthesizes research on the dual role of social media in shaping well-being among youth and explores how social-emotional learning (SEL) programs can be adapted to encourage a healthy relationship with digital platforms. Innovating SEL programs by incorporating educational techniques such as Problem-Based Learning, Inquiry-Based Learning, and Project-Based Learning inculcates real-world problem-solving and holds significant promise for fostering self-realization and self-regulation among the youth. By inculcating skills related to mindful technology use within SEL programs, students can learn to navigate social media and other technology related risk factors in a way that supports their mental health and overall well-being. The goal is to empower students to make informed decisions about technology and social media use, understand its effects on their mood and mental state, and practice techniques that contribute to a healthier, more balanced digital life. This holistic approach not only mitigates the risks associated with digital overuse but also leverages technology as a tool for enhancing personal and social development. Well-designed, innovative programs backed by thorough feasibility studies can help cultivate a generation of emotionally intelligent, resilient, and adaptive learners who can thrive in an increasingly complex digital landscape. Future research should focus on longitudinal studies to assess the long-term effects of these SEL innovations and develop scalable models that can be implemented across various educational settings.

## Introduction and background

Unmet need for prevention of mental health disorders in the youth

Several studies from various countries indicate that the COVID-19 pandemic has exacerbated psychological distress, anxiety, depression, drug misuse, and other mental health issues among youth [1, 2]. These widespread mental health challenges not only compromise student well-being but also have profound social, educational, and economic consequences, including academic underperformance and increased school and university dropout rates [3, 4].

The mental health of students in secondary and higher education has become an increasingly important issue, with estimates suggesting that one-fifth to one-third of this population may currently have a mental health condition, with many more at continual risk [5, 6]. Despite a significant rise in the demand for professional support, data reveals that just about a third of students experiencing mental health issues would seek help from counselling services [3]. Barriers such as stigma and a lack of awareness about available services prevent effective support access, potentially exacerbating these conditions [3].

In response to these escalating concerns, prominent organizations, the American Academy of Child and Adolescent Psychiatry, the American Academy of Pediatrics, and the Children’s Hospital Association, jointly declared a State of Emergency in Children's Mental Health precipitated by the pandemic, emphasizing the need for preventive measures [7]. Yet, the focus on interventions to prevent mental health disorders or promote resilience and mental well-being remains minimal compared to treatment, leading to inequity in health rights and expanding healthcare burden [8]. Additionally, poor mental health literacy is linked to poor mental health outcomes [3], which again highlights the need for mental well-being education and promotion with a focus on overall mental well-being rather than just the absence of mental health disorders. This approach acknowledges that mental health is more than the absence of illness and that promotion and prevention are interlinked, and enhancing well-being can prevent the onset of mental disorders [8].

Given that most lifetime mental disorders begin before adulthood, the most effective time for mental health education and inculcation is during childhood, adolescence, and emerging adulthood [9]. This crucial period allows for implementing evidence-based non-pharmacological interventions and psycho-educational programming. Educational institutions are pivotal in improving children’s mental health through the education system. Consequently, several think tanks and experts have called for a major reform of the educational system to provide better mental health education and support for youth [6, 10, 11]. The unrelenting need for effective social-emotional learning (SEL) interventions to foster resilience and enhance well-being is evident, as addressing mental health also improves student retention in higher education [4]. Research also indicates that social and emotional skills are crucial predictors of academic and career outcomes even after adjusting for socioeconomic status [12].

The key objective of this comprehensive narrative literature review is to explore the potential of innovating social-emotional learning (SEL) to enhance the positive engagement of youth with social media. The review investigates the key components of SEL that effectively promote positive mental health and well-being among youth. It also analyzes the dual role of social media in positively and negatively influencing the social and emotional development of youth. Furthermore, it identifies the most effective educational techniques that assist youth in regulating their behaviour through the conscious use of technology and social media. The adaptation of SEL programs to incorporate training on the mindful use of social media is also explored. Based on the gathered insights from the literature, we discuss the potential strategies for higher education institutions to develop and implement programs that foster a healthy relationship between students and social media. The citations for the discussion were retrieved through a semi-systematic search strategy using flexible keywords on two databases, PubMed and Google Scholar. This was complemented by searching cross-references from relevant articles to gather the most pertinent and useful citations related to the research topics. Research or analyses on the impact of different social media platforms were not within the scope of this review.

## Review

Social-emotional learning for boosting well-being in youth

Emotional intelligence has been shown to be a large indicator of success as an adult and in order to support its development we must focus on Social Emotional Learning (SEL) early in life [13]. Social-emotional development is described as the ability to understand and manage emotions, solve problems efficiently, and establish positive relationships [14]. It generally involves the process of imparting skills such as encoding, interpreting, and reasoning with social and emotional information [15]. The field of SEL has developed from research across various domains that are well-known to educators, researchers, and policymakers. This includes areas like social competence promotion, bullying prevention, drug use and abuse prevention, civic and character education, emotional intelligence, conflict resolution, social skills training, and other development of other skills related to emotional mastery [16].

Assessment of social and emotional skills presents conceptual and methodological challenges, with inconsistencies in frameworks, methodologies, and implementation practices potentially causing varied results in the evaluation of the effectiveness of SEL programs [12]. A recent meta-analysis reviewed the effectiveness of 12 high-quality SEL programs across 59 studies involving 83,233 participants [17]. The meta-analysis shows significant improvements in social-emotional skills, academic performance, and findings of prosocial behaviours, while reducing antisocial behaviours in the youth. Other effective strategies included enhancing teachers’ social-emotional capabilities and streamlining cognitive elements in SEL curricula. However, programs with insufficient durations were less effective. Another study showed that among various implementation factors, only the Quality of Delivery significantly impacted all evaluated outcomes. Hence, high-quality implementation and assessing implementation across multiple dimensions also seem necessary for achieving positive results with SEL [18].

Social media and its conflicting role as foe versus friend

Emerging data regards social media use and accompanying sedentary behaviours, multitasking, and disturbed sleep as key risk factors for escalating mental health issues among adolescents and young adults, necessitating robust interventions to safeguard their well-being in today’s digital environments [19, 20]. Although innovative programs are in the pipeline, most existing SEL curricula and techniques are not designed to address these risk factors associated with our modern life. Social media platforms play a significant role in adolescent development, offering spaces for growth, identity exploration, and social interaction. The EU Kids Online (EUKO) 2020 report from 19 countries highlights a substantial increase in internet use by adolescents compared with the survey findings from the 2010 report. The time kids spend online daily has nearly doubled in many countries, which also presents considerable risks [21]. An expert panel from a recent Delphi Study expressed significant concerns regarding the role of social media in amplifying pressures about physical appearance and exposing adolescents to negative behaviours [22]. Social media platforms often promote unrealistic beauty standards, such as athletic and muscular ideals for males and thin, curvaceous ideals for females, leading to objectified self-concepts and impossible body standards. The visual nature of social media, coupled with quantifiable peer feedback like likes and comments, intensifies these pressures during the sensitive period of adolescence [22]. Additionally, adolescents are especially vulnerable to cyberbullying and sexual harassment on these platforms [22]. Social media drug markets also substantially impact how young people access drugs by increasing availability and diminishing the age-related boundaries that traditionally separate social and commercial drug transactions [23]. However, a recent Pew Research Center survey of American teenagers aged between 13 and 17 years revealed mixed feelings about social media [24]. About 35% of teens valued social media as largely beneficial for fostering social connections and providing support during difficult times. Roughly one-third also admitted spending too much time on social platforms, and 54% confessed that they would find it somewhat difficult to give it up, with girls reporting more difficulty than boys. Additionally, as per the survey, the proportion of teens who report constantly being online has nearly doubled from 24% in 2014 to 46% in 2022, highlighting the massive influence of social media in their daily lives.

Jonathan Haidt's recent bestseller, *The Anxious Generation: How the Great Rewiring of Childhood Is Causing an Epidemic of Mental Illness*, blames smartphones and social media as the key factors in the rising mental health issues among youth [25].The book suggests that introducing age restrictions-14 years for smartphones and 16 years for social media could mitigate these problems. This stance aligns with the recent call from the United States (US) surgeon general for parental warning labels on social media to safeguard young minds, a proposal likely to face opposition from Big Tech [25]. With the importance of understanding the scientific evidence growing, Haidt’s book continues to dominate discussions, remaining a fixture on *The New York Times *Best Sellers list and receiving significant media attention [25].

However, some scholars have voiced scepticism, challenging the robustness of the evidence behind Haidt’s claims [25]. Research also supports the notion that, when used appropriately, social media can offer opportunities for social support, online companionship, and emotional intimacy. These elements are essential for healthy socialization and show that digital technology, including social media, can play a crucial role in facilitating meaningful social interaction among youth [26]. Social media could offer youth numerous educational benefits, enhancing formal and informal learning processes. Research shows that adolescents use social media to expand their knowledge and interests across diverse areas, such as reading, music, crafts, and gaming, often through affinity networks that provide coaching and support [27]. A large-scale study involving over 100,000 15-year-olds across 15 countries found that social media use correlates with improved online reading and information navigation skills [28]. Social platforms facilitate traditional educational activities like study groups and sharing homework [29], and even contribute to collaborative learning in science and engineering [30]. Additionally, a study in Spain highlighted that the informal learning environments created by social media enhance self-motivation and empower young learners [31]. Furthermore, using social media for academic writing has been linked to reduced writing anxiety and increased student agency in choosing writing topics [32], demonstrating the broad potential of social media to support and enhance youth education.

Capability building on mindful use of technology and social media

Overall, the relationship between social media use and psychological well-being is complex, manifesting both beneficial and detrimental effects [33]. Creating a digital environment that is secure, inclusive, engaging, and supportive for everyone necessitates avoiding reactions based on fear [34]. Effective self-regulation of social media usage can enhance psychological well-being by facilitating mood management, emotion-focused coping, and satisfying intrinsic needs. Conversely, poorly managed social media use can lead to goal conflicts, such as displacement and distraction from essential activities, ultimately harming well-being. Research indicates that failures in social media self-control consistently negatively impact mindfulness and subjective vitality [35]. While no complete reciprocal influence between social media self-control failure and mindfulness was found, partial interactions suggest that poor self-control in social media use could diminish mindfulness, potentially leading to a cyclical pattern of increased difficulties in regulating social media use [35].

A recent report from the National Academies of Sciences, Engineering, and Medicine in the US recommends creating new industry standards to limit the potential harms of social media on adolescent mental health while enhancing its benefits. The report highlights that, over the past 15 years, increased smartphone usage among young people has coincided with a decline in mental health [36]. The digital age has enabled billions to communicate via social media, yet it has also introduced significant challenges. One such challenge is the echo chamber effect, where closed systems amplify specific beliefs, leading young people to view social media as essential for survival. Unfortunately, a simple regulatory solution to address this growing social problem is not feasible [37]. Globally, an increasing number of countries are working on legally prohibiting the use of smartphones in schools and about one in seven countries now enforce such bans [38]. While many schools have successfully implemented these bans, once students leave school premises, they often have unrestricted access to these devices. Thus, merely banning phones at school does not eliminate all risks associated with their use [39]. Education on digital literacy and responsible social media use remain essential to mitigate potential harms.

Over the last decade, digital SEL interventions like virtual services, apps, virtual reality, and telemedicine have gained popularity due to their accessibility and convenience. However, comprehensive reviews on their effectiveness are limited. A recent systematic review indicates that digital tools have a moderate impact on improving mental health [40]. While these findings confirm their potential as valuable resources in mental health strategies, their primary role remains in managing mental health issues. There are also several targeted online mental health interventions for youth adopting cognitive behavioural therapy or family-based interventions that could enhance positive mental health indicators like well-being and resilience [41, 42]. However, these interventions are not specifically designed to foster self-awareness and regulation for optimal social media or technology use. Given the increasing challenges posed by digital and social media engagement and the critical role of self-regulation in socio-emotional competence, there is an urgent need to innovate SEL programs [43]. Comprehensive SEL programs need to aim to cultivate healthy digital habits, emphasizing the safe use of technology for personal development and mental health rather than allowing it to indulge in mindless scrolling or destructive comparisons with others. To address these issues effectively, SEL programs must integrate strategies that promote digital literacy and bring self-awareness and self-regulation in students to balance their online and offline lives and lead to responsible social media use.

Educational techniques on regulating behavior for conscious use of technology

Innovative SEL online and face-to-face programs can significantly benefit from incorporating interactive workshop style and student-led techniques such as Problem-Based Learning, Inquiry-Based Learning (IBL), and Project-Based Learning. These approaches, which emphasize real-world problem-solving and holistic inquiry, are not yet widely adopted in institution-wide SEL curriculums but offer considerable potential for fostering self-regulation and addressing risks like excessive social media use among students.

Problem-Based Learning centres on using real-world problems to develop critical thinking and problem-solving skills. Often used in clinical education and implementation science, this learning technique can help students navigate their mission, purpose, goals and personal priorities and identify how these could conflict with their current engagement with social media, enhancing their decision-making and self-regulation skills [44-46]. By engaging with real-life challenges, students can learn to apply emotional intelligence in varied situations, promoting a deeper understanding and mastery of using technology for their personal development [47]. An example of using this learning method would be to ask students, after a brief tutorial, to rate their sleep quality and create a personalized sleep plan that incorporates best practices to avoid the impact of devices on sleep (e.g., keeping devices away from the bedroom or furthest away from the bed). In designing their sleep plan, students engage in active problem-solving by reflecting on their habits and applying best practices. They will need to assess their current device usage, identify challenges to proper sleep hygiene, and devise practical strategies for improvement (Table 1).

**Table 1:** Activities for enhancing mindful use of social media across learning techniques Activities across learning techniques may overlap depending on how they are designed, structured, and implemented. Educators should also consider the age, maturity, cultural background, and other social factors of their students while choosing the activity for each technique.

Learning technique	Activity	Description
Problem-Based Learning	Digital footprint analysis	Students map out their social media activity to identify current risks and conflicts with personal values and goals. Students engage in active problem-solving by reflecting on their habits. Students track and manage their social media usage to balance it with academic and personal goals.
	Personalized sleep plan	Students rate their sleep quality and create a personalized sleep plan Students assess their current device usage, identify challenges to proper sleep hygiene, and devise practical strategies for improvement. The plan incorporates best practices to avoid the impact of devices on sleep.
Inquiry-Based Learning	Mood tracking reflection	Students document and analyze their emotions before and after social media use to identify patterns.
	Content curation exploration	Students curate their feeds with motivational and educational content types and observe the effects on their well-being.
Project-Based Learning	Social media detox	Students design and implement a personal or group plan that incorporates a temporary social media break or restricts its use to educational purposes only.
	Character simulation presentations	Students develop a detailed profile of a fictional individual, including their interests, and daily routines. They illustrate the differences in the character’s life outcomes without and with mindful use of social media habits.

Inquiry-Based Learning (IBL) adopts a more flexible approach. It encourages students to lead their investigative processes, fostering a sense of curiosity and autonomy [48]. IBL encourages students to reflect on the impact of their lifestyle preferences on emotional responses and guide appropriate behaviour [49, 50]. This method also allows students to explore and enhance their emotional intelligence, which is crucial for personal development and self-regulation. Examples of IBL exercises include asking students to reflect on how social media use affects their emotional responses and guiding them to explore the connection between their social media habits and their emotional states. Another exercise could be framing a question like this: ‘Suppose you are mentoring a younger teen who has just received their first phone. What advice would you give?’ It could also be in the form of guided inquiry, e.g., by altering social media feeds to include mostly motivational or educational content and asking students to explore the relationship between the content they consume and their personal growth (Table 1).

Project-Based Learning immerses students in real-world and personally meaningful projects. This approach is significant for context-based curriculum education as it emphasizes the importance of context in learning and connects academic concepts to real-life applications [51]. By working on projects related to mindful use of social media, students can engage more deeply with the material and develop a broader range of competencies, including higher-order thinking skills such as critical thinking, reflective thinking, creative thinking, dialogic/dialectic thinking, decision-making, problem-solving, and emotional intelligence [52]. Examples include designing a social media detox plan, a public awareness campaign or a presentation on the responsible use of social media. Another example could be group projects, such as creating a character and depicting their life with and without mindful social media use (Table 1). It is important to note that activities across various learning techniques may intersect and complement one another, depending on how they are designed, structured, and implemented. This overlap allows educators to choose and integrate multiple approaches, depending on their preferences and expertise, as well as the age, maturity, cultural background, and other social factors of their students.

**Figure 1:**
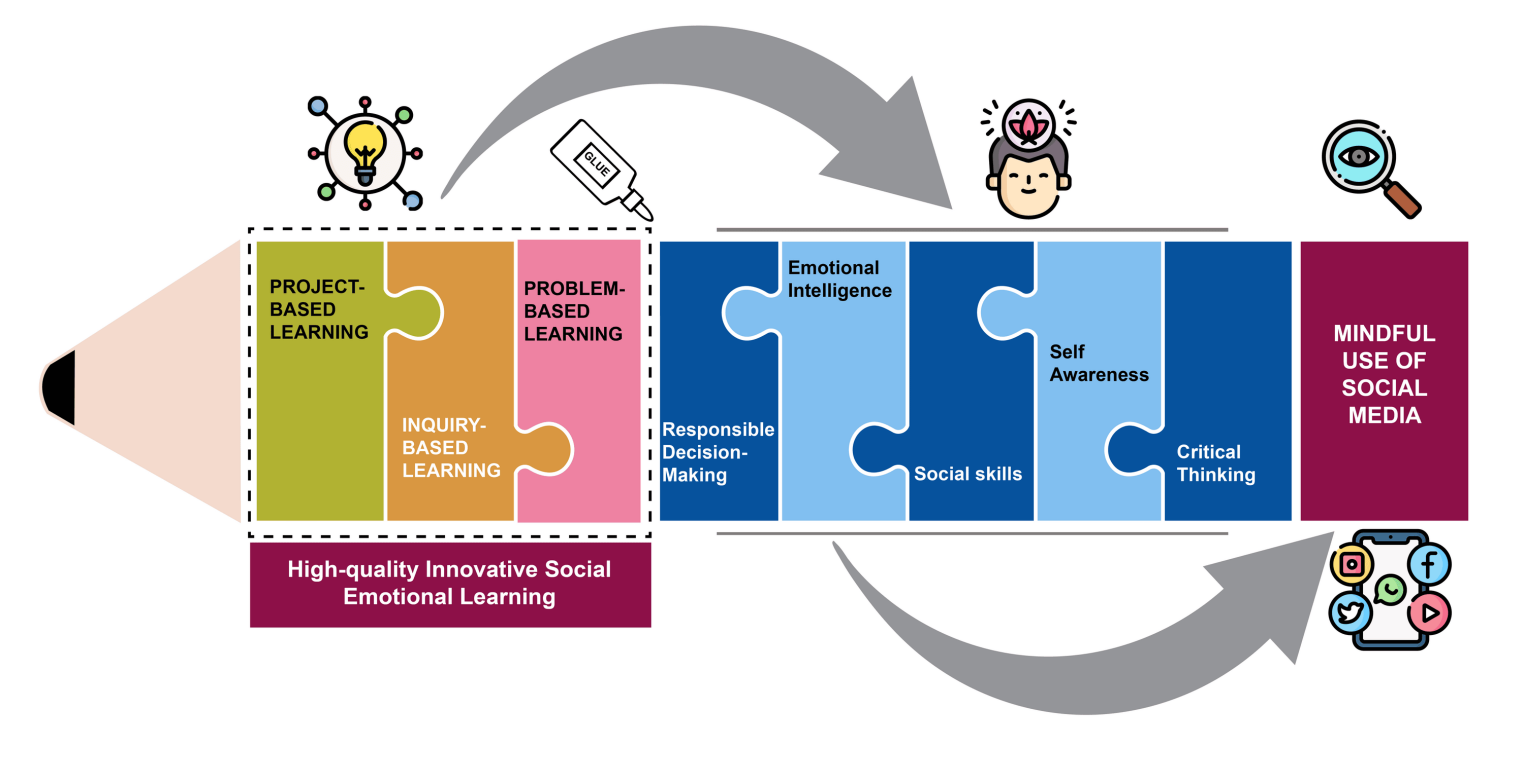
Proposed SEL framework incorporating student-led educational techniques for enhancing positive engagement and mindful use of social media by youth The figure illustrates a model for fostering mindful social media use among youth by integrating student-led educational techniques with social-emotional learning (SEL). The framework also highlights three educational methodologies: Problem-Based Learning, Inquiry-Based Learning, and Project-Based Learning, which serve as the mechanisms to apply SEL principles effectively. This educational approach aims to glue together different learning styles and strategies to promote high-quality, innovative SEL that can positively impact students' interaction with social media. Incorporating interactive educational techniques is necessary to impart essential SEL competencies such as critical thinking, self-awareness, social skills, emotional intelligence, and responsible decision-making to students, which are crucial elements for enhancing mindful engagement with social media. Image credits: This image was created by the corresponding author (Sajita Setia).

Figure 1 illustrates a model that integrates student-led educational techniques with SEL to foster mindful social media use among youth, highlighting the relationship between these educational techniques to effectively apply SEL principles, which are essential for enhancing positive engagement with social media. Integrating these methodologies within an outcomes-based education framework is also necessary to ensure that learning is competency-driven, focusing on specific outcomes that learners are expected to achieve. This approach aligns educational objectives with the skills needed in the digital age, including critical analysis, effective communication, and ethical decision-making regarding technology use.

Implications and future directions

Although the prevention of cardiovascular diseases and cancers frequently dominates policy discussions and service delivery, the prevention of mental illness is often overlooked [53]. Evidence indicates that it is unrealistic to expect mental health professionals alone to shoulder the responsibilities of promoting and preventing mental health issues [54]. This timeline for the development of mental health disorders offers numerous opportunities for intervention in schools that largely cater for education with the aid of technology for the prevention of declining mental health due to the abuse of digital devices. Therefore, it is vital to develop reliable and effective methods to transform the growing evidence base on mental illness prevention into practical policy recommendations, moving the prevention agenda forward effectively. Educational institutions should consider developing a robust curriculum that integrates these methodologies into SEL frameworks. Such a curriculum should focus on real-world issues relevant to students' lives, including managing digital assignments while being mindful of the impact of heavy digital life and social media on mental health and developing healthy online and offline relationships. These would need feasibility studies on comprehensive interventions, eventually aiming for whole-school approaches provided the programs are practical and sustainable with modest resource investment [55]. Training routine staff such as teachers, school counsellors, and peers as instructors for SEL is advocated for sustainable and cost-effective implementation. However, this strategy introduces new challenges regarding the collaboration between professionals and mentors, the additional skills required by trainers and funding sources for these programs [56]. Nevertheless, conducting carefully planned pilot feasibility studies at schools and universities could pave the way for identifying optimal models for large-scale, evidence-based interventions. Higher education institutions should prioritize mental health, seeking grants and investments to support the well-being of future leaders. Ensuring universal access to preventive mental health interventions is as vital as maintaining continuous educational opportunities for all students. Moreover, collaborative efforts across sectors are necessary to guarantee equitable access to mental health resources, overcome disparities, and implement lasting solutions that enhance the mental health of children, adolescents and emerging adults in this post-pandemic period.

## Conclusions

There is an urgent need for industry standards to mitigate the adverse effects of social media on adolescent mental health. This need arises as increased smartphone usage over the past 15 years correlates with declining mental health, intensified by the echo chamber effect in social media. Transforming social media from a foe to a friend involves leveraging its potential to enhance communication and education while mitigating its risks to mental health. Integration of problem-based, inquiry-based, and project-based learning related to mindful use of technology into SEL frameworks holds great potential to mitigate this increasingly complex social issue.

To effectively implement these strategies, schools should undertake feasibility studies with innovative SEL incorporating digital mindfulness capability building for sustainable approaches with modest resources. Training teachers, school counsellors, and peers as SEL instructors is crucial for a cost-effective rollout despite challenges in collaboration and funding. Moreover, prioritizing mental health through grants and collaborative efforts across sectors is essential to ensure universal access to mental health interventions. Such comprehensive measures aim to cultivate a generation of emotionally intelligent, resilient, and adaptable learners who can excel in an increasingly complex digital landscape.
